# Optimal chest compression technique for paediatric cardiac arrest victims

**DOI:** 10.1186/s13049-015-0118-y

**Published:** 2015-04-22

**Authors:** Min Joung Kim, Hye Sun Lee, Seunghwan Kim, Yoo Seok Park

**Affiliations:** Department of Emergency Medicine, Yonsei University College of Medicine, 50 Yonsei-ro, Seodaemun-gu, Seoul, 120-752 Republic of Korea; Department of Biostatistics, Yonsei University College of Medicine, Seoul, Republic of Korea

**Keywords:** Cardiopulmonary resuscitation, Fatigue, Hand, Heart arrest, Heart massage, Paediatrics

## Abstract

**Background:**

The aim of this study was to assess the quality of chest compressions performed by inexperienced rescuers using three different techniques: two-hand, right one-hand, and left one-hand.

**Methods:**

We performed a prospective, randomised, crossover study in a simulated 6-year-old paediatric manikin model. Each participant performed 2-minute continuous chest compressions, using three different techniques. Chest compression quality data, including compression rate, compression depth, and residual leaning was recorded by a Q-CPR™ compression sensor connected to HeartStart MRx (Philips Healthcare, Andover, MA, USA). To examine trends in chest compression performance over time, each 2-minute period was divided into six consecutive 20-second epochs.

**Results:**

The 36 participants completed 108 two-minute trials, consisting of a total of 25,030 compressions. The mean compression rates [95% confidence interval] were as follows: two-hand, 116.8 [111.7–121.9]; left one-hand, 115.0 [109.9–120.1]; and right one-hand, 115.5 [110.4–120.6] (*p* = 0.565). The mean compression depth for two-hand was 38.7 mm (37.1–40.2), which was higher than for left one-hand (36.3 mm [34.8–37.9]) or right one-hand (35.4 mm [33.9-37.0]) (*p* < 0.001). Chest compression depth declined over time, regardless of the technique (*p* < 0.001). The pattern of compression depth change over time was similar for all techniques (*p* > 0.999). The residual leaning rate was higher with two-hand (40.7 [27.9–53.5]) than that for left one-hand (29.2 [16.4–42.0]) or right one-hand (25.8 [13.0–38.6]) (*p* = 0.021).

**Conclusions:**

For paediatric cardiopulmonary resuscitation by inexperienced rescuers, the two-hand technique has the advantage of producing deeper compressions than the one-hand technique, but it is accompanied by more frequent residual leaning. For the one-hand techniques, the right and left hand produced chest compressions of similar quality.

## Background

The incidence of paediatric out-of-hospital cardiac arrest (OHCA) in the general population is 8.0 to 19.7 per 100,000 person-years [1-4]. Considering the loss of potential years of productive life, the importance of successful resuscitation of paediatric victims cannot be overemphasized, although paediatric OHCA is an infrequent event.

In paediatric cardiopulmonary resuscitation (CPR), high-quality chest compressions are essential to successful resuscitation. The 2010 European Resuscitation Council Guidelines and American Heart Association guidelines emphasize deeper (at least one third the anterior–posterior (AP) diameter of the chest) and faster (at least 100 compressions per minute) chest compressions, with complete chest recoil after each compression [5,6]. These guidelines further indicate that rescuers may use either a one-hand or two-hand compression technique for a child victim, thereby allowing rescuers to adapt the technique to the victim’s size and rescuer’s strength. The one-hand technique has also been recommended for children based on the suppositions that less force is required for paediatric chest compressions and that the one-hand technique is associated with a lower risk of internal organ injury. However, the guideline that allows for two different compression methods for children might lead to confusion among inexperienced lay rescuers. Also, teaching both techniques may contribute to poor skill acquisition of basic life support among the general public.

Evidence supporting these suppositions is lacking, and there have been few comparative studies to determine which compression method is most likely to provide high-quality chest compressions [7-9]. These child manikin studies suggested that the two-hand technique generated higher compression pressures [8,9], and compression rate decreased faster with a one-hand technique [7]. However, other quality indicators of chest compressions, such as compression depth and complete chest recoil, were hardly investigated. Furthermore, the participants of all previous studies were well-trained healthcare providers; therefore, their results cannot be applied to inexperienced laypersons. Additionally, no study has assessed compression quality of the one-hand technique comparing the right and left hands.

Hence, the aim of this study was to assess whether the quality of chest compressions as defined by adherence to current guidelines performed by inexperienced rescuers in a paediatric manikin model differed between three techniques: right one-hand, left one-hand, and two-hand. We hypothesized that the quality of chest compressions using a two-hand technique would be superior to the quality of compressions using a right or left one-hand technique, and using the dominant hand would have an advantage over using the non-dominant hand.

## Methods

### Study design and participants

We performed a prospective, randomised, crossover study approved by our Institutional Review Board (IRB). A total of 36 medical students, who had received CPR training within the past 1 year based on current guidelines, were recruited voluntarily over 6 months, using IRB-approved study posters throughout the Yonsei University College of Medicine. Written informed consent was obtained from all participants. Demographic information, including age, gender, weight, height, and dominant hand, was recorded. We excluded participants with previous experience in performing CPR in real situations or with any medical condition that contraindicated the physical exertion required for CPR.

### Study protocol

Before beginning the study, each participant received a 5-minute demonstration with instructions about performing one-hand and two-hand chest compressions on children. During these instructions, high-quality chest compressions defined by deeper and faster compressions and complete chest recoil were also emphasised. The participants were blinded about the type of data being recorded. They were allowed to have a practice session to familiarise themselves with performing chest compressions using the Q-CPR^TM^ compression sensor connected to HeartStart MRx (Philips Healthcare, Andover, MA, USA). This equipment recorded chest compression quality parameters, such as compression rate, compression depth, and residual leaning. All compressions were performed on Little junior^TM^ (Laerdal Medical, Stavanger, Norway), a 6-year-old child manikin, with an AP diameter of 5.5 inch (14.0 cm). During compressions, the participants kneeled on the floor beside the manikin, to mimic the conditions of out-of-hospital CPR.

Each participant performed 2-minute continuous chest compressions without ventilation using the three techniques: right one-hand, left one-hand, and two-hand. Between each 2-minute period of compressions, the participant rested for 10 minutes to minimise fatigue. To avoid potential bias, no audiovisual feedback was provided to the participants during the experimental session. The order in which the three compression techniques were performed by each participant was randomly assigned by a randomization code using permuted block randomization for Williams 6*3 (sequence*method) crossover design [10].

Chest compression quality data, including compression rate, compression depth, and residual leaning, were collected and analysed using the HeartStart Event Review Pro Hospital Edition, Version 4.1.2 (Laerdal Medical, Stavanger, Norway). Residual leaning was defined as ≥2.5 kg residual force on the chest at the end of the release phase of each compression. The residual leaning rate was defined as the ratio of the number of compressions with residual leaning to the total number of compressions. To examine trends in chest compression performance over time, each 2-minute chest compression period was divided into six consecutive 20-second epochs. All chest compressions were recorded with a video recorder, and all trials were reviewed to count the number of times the hand(s) slipped on the compression sensor. At the end of the experimental session, participants used a visual analogue scale (0 mm, extremely easy to 100 mm, extremely difficult) to rate the subjective difficulty of chest compressions with each technique. Before and after the experimental session, the participants were asked which compression technique they preferred. The primary endpoints were mean chest compression rate and depth. Additional endpoints included the residual leaning rate, the number of slipped compressions, and the subjective degree of compression difficulty. Data were extracted by one investigator (MJK), who was blinded to the compression technique performed.

### Statistical analysis

All statistical analyses were performed using SAS 9.2 (SAS Institute INC., Cary, NC, USA). The sample size power was calculated according to our primary endpoints. Based on Udassi’s report [9], we chose 125 compressions/min as the mean compression rate and 32.9 mm as the mean depth of chest compression, using the two-hand technique. Differences between the three techniques of 10 compressions/minute and 5 mm mean compression depth (assuming a standard deviation of the differences of 20 and 10, respectively) were selected as the minimum clinically significant values. Using these values, we calculated that a sample size of 6 participants in each sequence group would be adequate, at a significance level of 0.05 (two sided) with 80% power [11]. The total sample size was thus 36.

Discrete variables are presented as rate (%) and continuous variables as mean ± SD. The chest compression performance variables and subjective degree of difficulty were analysed using a linear mixed model for Williams crossover design. Three fixed effects were included: period effect, sequence effect (between-subject effect), and group effect (within-subject effect). Differences in the preferred chest compression technique between before and after the trial were analysed using a generalized McNemar’s test. A linear mixed model was also used to compare trends in chest compression performance over time. Fixed effects were compression technique (between-subject effect), time (within-subject effect), and compression technique by time interaction. Additionally, we performed *post hoc* analyses to estimate the time points at which the compression depth significantly declined between 20-second epochs. In the *post hoc* analysis, the least square means of the three techniques were estimated at each epoch and compared by a linear mixed model. A *p* value less than 0.05 was considered statistically significant and the significance level was adjusted using the Bonferroni correction for multiple comparisons for the *post hoc* analysis.

## Results

### Patient demographics

All 36 participants successfully completed the study. Their mean age was 25.9 ± 2.0 years, and 23 (63.9%) participants were male. Other demographic characteristics are presented in Table 1. Upon analysis using a linear mixed model, the mean compression rate, compression depth, and number of slipped compressions were not affected by demographic characteristics, including participant height (data not shown). However, height was independently associated with the residual leaning rate. The residual leaning rate increased by 1.4% per 1.0 cm increase of the height (*p* = 0.033)

**Table 1:** **Demographics of study participants (N = 36)**

**Variable**		**Rate (%) or Mean ± SD**
Age (year)		25.9 ± 2.0
Gender, n (%)	Male	23 (63.9)
	Female	13 (36.1)
Height (cm)		169.6 ± 9.2
Weight (kg)		60.8 ± 10.1
BMI (kg/m^2^)		21.0 ± 1.9
BSA (m^2^)		1.7 ± 0.2
Dominant hand, n (%)	Right	35 (97.2)
	Left	0
	Both	1 (2.8)

SD = standard deviation, BMI = body mass index, BSA = body surface area.

### Overall chest compression quality

Data from 108 two-minute trials, consisting of a total of 25,030 chest compressions, were analysed. The mean compression rate (95% confidence interval) was 116.8 compressions/minute (111.7–121.9) using the two-hand technique, 115.0 compressions/minute (109.9–120.1) using the left one-hand technique, and 115.5 compressions/minute (110.4–120.6) using the right one-hand technique. These numbers remained within the current guidelines of ≥100 compressions/min for all techniques throughout the 2-minute trials. Although a significant period effect was observed (with an increase in compression rate during the later period of compression), the compression rate did not differ among techniques after statistical correction (*p* = 0.565). The estimated mean compression depth was 38.7 mm (37.1–40.2) using the two-hand technique, 36.3 mm (34.8–37.9) using the left one-hand technique, and 35.4 mm (33.9–37.0) using the right one-hand technique (*p* < 0.001). *Post hoc* analysis indicated that the compression depth using the two-hand technique was greater than the depth for the left or right one-hand technique (*p* = 0.002 and *p* < 0.001, respectively), but the compression depth did not differ between the left one-hand and right one-hand techniques (*p* = 0.215). However, most participants did not achieve the recommended depth of ≥50.0 mm for any compression technique. The residual leaning rate was higher with the two-hand than with the left one-hand or right one-hand technique (*p* = 0.021). This difference remained after correcting for the association between the participants’ height and residual leaning rate. The number of times the hand(s) slipped did not differ among techniques (Table 2).

**Table 2:** **Comparison of chest compression quality and the subjective degree of difficulty among the three techniques**

**`**	**Chest compression methods**			***P*** **-value for mixed model**		
	**Two-hand**	**Left one-hand**	**Right one-hand**	**Sequence**	**Period**	**Method**
Compression rate (compressions/min)	116.8(111.7-121.9)	115.0(109.9-120.1)	115.5(110.4-120.6)	0.346	<0.001*	0.565
Compression depth (mm)	38.7(37.1-40.2)	36.3(34.8-37.9)	35.4(33.9-37.0)	0.414	0.054	<0.001*
Residual leaning rate (%)^1^	40.7(27.9-53.5)	29.2(16.4-42.0)	25.8(13.0-38.6)	0.645	0.056	0.021*
Number of slipped compressions	0.6(-0.2-1.3)	1.5(0.8-2.2)	0.7(-0.1-1.4)	0.102	0.194	0.097
Subjective difficulty (mm) ^2^	60.8(55.7-66.0)	72.4(67.3,77.5)	70.0(64.8-75.1)	0.147	0.789	<0.001*

All values are estimated means (95% confidence interval) except *p* values.

^1^the ratio of the number of compressions with residual leaning to the total number of compressions; residual leaning is defined as ≥2.5 kg residual force on the chest at the end of the release phase of each compression.

^2^rated as visual analogue scale (0 mm, extremely easy to 100 mm, extremely difficult).

*p < 0.05 is considered to be statistically significant.

### Compression quality over time

**Figure 1:**
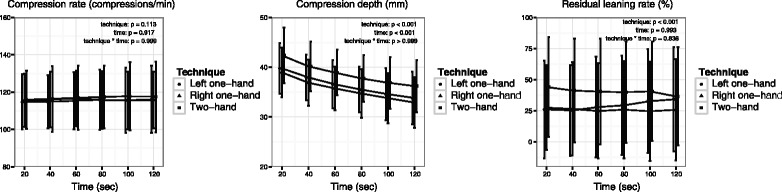
Trends in chest compression performance over time. Circles and bars represent means and standard deviations, respectively. Residual leaning rate is the ratio of the number of compressions with residual leaning to the total number of compressions; residual leaning is defined as ≥2.5 kg residual force on the chest at the end of the release phase of each compression.

After dividing the data into 20-second epochs, a total of 648 epochs were analysed. No compression technique by time interaction was observed for the compression rate, suggesting that the pattern of change in compression rate over time did not differ among the three techniques (*p* = 0.999). There was also no difference in the compression rate considering time or technique effects (*p* = 0.917 and *p* = 0.113, respectively). The pattern of change in the compression depth over time was similar among the three techniques (*p* > 0.999). The chest compression depth per 20-second epoch declined over time for all techniques (*p* < 0.001) (Figure 1). *Post hoc* analyses indicated that for every compression technique, the greatest compression depth decline occurred between the first and second epoch (two-hand, *p* = 0.003; left one-hand, *p* = 0.014; and right one-hand, *p* = 0.005, respectively). No interaction or time effect was observed for the residual leaning rate (*p* = 0.838 and *p* = 0.993, respectively).

Participants’ Opinions Regarding Difficulty and Preferred Compression Technique

The participants reported that the two-hand technique was less difficult than the other techniques (*p* < 0.001) (Table 2). Before the trials, the preferred method was two-hand for 28 (77.8%) participants and left one-hand for eight (22.2%) participants. After the experimental session, the preferred technique was two-hand for 30 (83.3%) participants, right one-hand technique for four (11.1%) participants, and left one-hand technique for two (5.6%) participants. These before and after preferences were not significantly different (*p* = 0.343).

## Discussion

Paediatric OHCA is generally considered to have a poor prognosis, and resuscitative efforts for paediatric arrest victims have often been assumed to be futile [4]. However, recent studies have reported that outcomes of children are better than those of adults after OHCA [1,12], and the importance of performing high-quality chest compressions during paediatric CPR has been emphasised [13]. Only a few reports have heretofore compared the quality of chest compressions using two-hand and one-hand techniques [7-9], and no study has compared the qualities of right and left one-hand chest compressions.

In general, the younger and smaller the child, the faster their normal heart rate. Previous studies reported similar compression rates for one-hand and two-hand techniques [7,9], but compression rates fell faster with a one-hand technique [7]. However, a recent study reported that the number of chest compressions per 30-second epoch did not decline over a 2-minute study period during simulated paediatric CPR performed by highly trained personnel with CPR experience [14]. Unfortunately, the authors did not describe the compression technique. Our study showed no differences in compression rate among the three techniques, which remained within the recommended range of ≥100 compressions/min throughout the 2-minute test period, and no change in the compression rate over time for any technique. This indicates that all three techniques allow inexperienced rescuers to not only achieve the recommended compression rate but also maintain this rate for 2 minutes of continuous compressions.

Our study demonstrated that the compression depth using the two-hand technique was greater than that of the left or right one-hand technique. These results are consistent with those of two previous simulated paediatric resuscitation studies. Stevenson et al. reported that two-hand chest compressions produced significantly higher mean and peak pressures than one-hand compressions [8]. Udassi et al. likewise showed a trend towards higher compression depth and peak compression pressure with the two-hand technique, although the difference between two-hand and one-hand techniques was not statistically significant [9]. In this study, the depth difference between one-hand and two-hand techniques was 2.4–3.3 mm. As there is no study that has identified the correlation of compression depth with treatment outcome in paediatric victims, it is difficult to determine the clinical significance of our result. For adult victims, Vadeboncoeur et al. reported that each 5-mm increase in mean chest compression depth significantly increased both survival in general and survival with favorable functional outcome, with an odds ratio of 1.29 and 1.30, respectively [15]. Our study also showed that compression depth continued to decline over time during the 2-minute trial period, although the decline was most prominent between the first and second epoch. The pattern of compression depth decay over time was comparable for all techniques, thereby suggesting that rescuer fatigue affected all techniques similarly. Udassi et al. reported no significant differences between two-hand and one-hand techniques for compression depth or peak compression pressure over time during simulated 5-minute paediatric CPR [9]. One possible explanation for the difference between their results and ours is that unlike the previous research, our participants were inexperienced rescuers who were not as capable of detecting deterioration in their technique due to lack of experience, and so may not attempt to compensate as much. With the manikin used in our study, a compression depth of 46.7 mm (one third the AP diameter) should be achieved to meet the current guidelines. However, we did note that almost all participants failed to achieve the recommended chest compression depth during every technique. Although our inexperienced participants may not be capable of compressing to a sufficient depth, other studies also showed that well-trained health care practitioners could not achieve the recommended depth in child manikins [9,14,16,17]. This implies that child manikins may differ from real children in resistance and stiffness of the chest wall.

Complete chest recoil is another important aspect of high-quality CPR [18-20]; however, the influence of compression method on residual leaning rate has not been investigated. In this study, the residual leaning rate was higher with the two-hand technique than the left or right one-hand techniques. The posture of the two-hand technique with both arms forming an isosceles triangle will aid rescuers in loading their weight on the chest wall comfortably during each compression. Although this posture is helpful to achieve deeper compression, the risk of incomplete recoil would be increased. Interestingly, participant height was also associated with the residual leaning rate, and there was also a tendency for height to correlate inversely with the amount of recoil allowed. This result is consistent with that of a previous study in which the authors reported that rescuers >170 cm in height exhibited significantly more residual leaning than rescuers <170 cm [21].

We used the Q-CPR™ compression sensor on the manikin’s chest for recording chest compression quality parameters, and the hand position of the participants was predetermined on the sensor. Therefore, we could not determine whether the techniques differed in their ability to maintain the correct position of the hand(s). Instead of directly evaluating correct hand position, we recorded the number of times the hand slipped on the sensor. In Peska’s study, 65.6% of participants preferred the two-hand compression technique, partly because it was easier to maintain their balance with that technique [7]. However, our results did not demonstrate a difference among techniques for the number of times the hand(s) slipped. Our participants did think that performing chest compressions using the two-hand technique was easier than the other techniques. Unfamiliarity with the one-hand technique might have contributed to its subjective difficulty. Also, for such a reason as mentioned, the majority of participants favoured the two-hand compression technique both before and after the experimental session, which was consistent with the findings of previous studies [7,8].

Handedness is the tendency to consistently favour the use of one hand/arm for performing selected tasks. In general, dominant arm performance is better for activities requiring precision of inter-joint coordination (e.g., cutting paper with scissors), whereas non-dominant arm performance is more specialized for control of steady-state limb position (e.g., holding a piece of paper for cutting) [22-24]. Contrary to our expectations, chest compression quality was similar for the right and left one-hand techniques. This suggests that arm dominance does not affect compression depth and rate. Sainburg and Kalakanis reported that during rapid targeted reaching movements, the right and left hands showed a similar time course of improvement in final position accuracy over repeated trials, and the final accuracy was similar for both hands after task adaptation [25]. These findings are consistent with our results. Indeed, performance of chest compressions may be affected by multiple factors, such as age, gender, and muscle power of the rescuer, in addition to handedness.

There were limitations to this study. First, the study used a child manikin to simulate paediatric cardiac arrest. Although various child manikins are widely used in paediatric CPR training and studies investigating CPR performance, they may not exactly replicate the characteristics (e.g., stiffness, resistance) of the paediatric thorax. Therefore, our findings may not be applicable to actual clinical settings. Additionally, our use of only a 6-year-old child manikin indicates that our results may not reflect chest compression performance on children of all ages. Second, our use of the Q-CPR^TM^ compression sensor to record chest compression quality parameters may have affected the results, as the participants were not familiar with performing compressions on it. However, we attempted to minimise this confounding factor by allowing a practice session before starting the experimental session. Third, most participants were right-handed (as are most Koreans), so we were unable to examine the effect of handedness on chest compression quality. The participants were also young and healthy; thus, our results may not be generalisable to other rescuers. However, most parents are also young and healthy, and the benefits of a two-hand technique may be better suited for older and less healthy individuals who may have more difficulty providing the compression force required for the one-hand technique. Fourth, although the medical students who participated in the study were inexperienced in real-world CPR, they would have had more knowledge of resuscitation, anatomy, and physiology than a normal population. Additionally, our participants were more likely to be enthusiastic and confident, considering that they all actively responded to posters advertising the study. These characteristics of our participants hinder generalization of our results to inexperienced rescuers. Fifth, participants were aware that they were being evaluated and videotaped. This may have led to the Hawthorne effect, in which the participants performed better than they would in a real-life situation. Conversely, they may have performed compressions less effectively because no actual child required resuscitation. Nevertheless, except for the issue of the participants’ handedness, it is likely that these limitations would have affected chest compression performance similarly for all techniques. Finally, we did not include ventilation despite the fact that ventilation is more important in asphyxia-induced arrest (the most common etiology in paediatric victims). We do acknowledge that this was a simulation study limited to the investigation of compression and suggest that further studies are needed to explore these limitations in a clinical context.

In this study of simulated paediatric CPR performed by inexperienced rescuers, we demonstrated that the mean compression rate did not differ among three chest compression techniques, nor did the compression rate change over a 2-minute period of continuous chest compressions for any technique. The mean compression depth using the two-hand technique was greater than that of the left or right one-hand technique, but most participants did not achieve the recommended depth with any technique. Moreover, the chest compression depth declined over time during all three techniques. The incomplete chest recoil occurred more frequently with the two-hand technique than with the one-hand technique. The performance of chest compressions was similar for the right and left one-hand techniques. These findings are important for instructors as well as for team members who watch CPR quality during actual resuscitation. By knowing tendencies of different techniques, they can use this information to recognise these specific problems with performance.
